# Consistency of sugar structures and their annotation in the PDB

**DOI:** 10.1186/1758-2946-6-S1-P41

**Published:** 2014-03-11

**Authors:** Deepti Jaiswal, David Sehnal, Radka Svobodová Vařeková, Crina-Maria Ionescu, Jaroslav Koča

**Affiliations:** 1 National Centre for Biomolecular Research, Faculty of Science and CEITEC -Central European Institute of Technology, Masaryk University, Brno, 625 00, CZ, Czech Republic

## 

Cell-cell recognition is the first stage in many important phenomena such as infection by bacteria and viruses, communication among cells of lower eukaryotes, binding of sperm to egg, etc. [1]. Cell-cell recognition relies on sugar (carbohydrate) specific interactions at the cell surface. Theoretical studies typically involve molecular modeling of sugars and sugar-specific protein receptors. These studies rely on structural information obtained mainly by crystallography and nuclear magnetic resonance, and deposited in the Protein Databank (PDB). Since the main purpose of PDB is to store the structure of proteins and nucleic acids, thus, it is expected that PDB structure files are complete and correctly annotated.

Nonetheless, sugars exhibit a structural diversity larger than amino acids or nucleotides, a property which makes them ideal for recognition. At the same time, sugars are characterized by specific and very sensitive structural features such as multiple chiral centers on each ring. Because of these peculiarities, the validation and annotation of sugar structures is not straightforward.

Our first goal was to develop a methodology that can identify whether a sugar structure is complete and correctly annotated. Our second goal was then to check all PDB entries containing sugars, and record whatever problems we encounter in the sugar structures. For this purpose we collected all sugar structures which appear as ligands in PDB entries, and compared them to model structures available in Ligand Expo [2], a curated repository of ligand chemical and structural information. In order to perform the comparison we used several tools for structural comparison currently available (SiteBinder [3], Open Babel [4]), as well as two in-house programs. We report here on our findings regarding the complete and correctly annotated sugar structures in PDB, together with the problematic cases.
